# Terbium and Europium Chlorocyananilate-Based 2D Coordination Polymers

**DOI:** 10.3390/molecules28186453

**Published:** 2023-09-06

**Authors:** Mariangela Oggianu, Alexandre Abhervé, Daniela Marongiu, Francesco Quochi, José Ramón Galán-Mascarós, Federica Bertolotti, Norberto Masciocchi, Narcis Avarvari, Maria Laura Mercuri

**Affiliations:** 1 Department of Chemical and Geological Sciences, University of Cagliari, Highway 554, Crossroads for Sestu, I-09042 Monserrato, Italy; mariangela.oggianu@unica.it; 2 National Interuniversity Consortium of Materials Science and Technology, INSTM, Street Giuseppe Giusti, 9, I-50121 Florence, Italy; quochi@unica.it (F.Q.); norberto.masciocchi@uninsubria.it (N.M.); 3 Laboratoire MOLTECH-Anjou UMR 6200, UFR Sciences, CNRS, Université d’Angers, Bât. K, 2 Bd. Lavoisier, I-49045 Angers, France; alexandre.abherve@univ-angers.fr; 4 Department of Physics, University of Cagliari, Highway 554, Crossroads for Sestu, I-09042 Monserrato, Italy; dmarongiu@dsf.unica.it; 5 Institute of Chemical Research of Catalonia (ICIQ-CERCA), Barcelona Institute of Science and Technology (BIST), Avenida Països Catalans 16, 43007 Tarragona, Spain; jrgalan@iciq.es; 6 ICREA, Av. Lluis Companys 16, Passeig Lluís Companys, 23, 08010 Barcelona, Spain; 7 Dipartimento di Scienza e Alta Tecnologia & To.Sca.Lab., University of Insubria, Via Valleggio 11, I-22100 Como, Italy; federica.bertolotti@uninsubria.it

**Keywords:** lanthanides, 2D coordination polymers, nanosheets, anilates

## Abstract

Two-dimensional layered coordination polymers based on the hetero-substituted 3-chloro-6-cyano-2,5-dihydroxybenzoquinone ligands, hereafter ClCNAn^2−^ anilate, and Ln^III^ ions (Tb and Eu) are reported. Compounds **1** and **2**, formulated as Ln_2_(ClCNAn)_3_(DMSO)_6_ (Ln^III^ = Tb, **1**; Eu, **2**), and their related intermediates **1′** and **2′**, formulated as Ln_2_(ClCNAn)_3_(H_2_O)_x_·yH_2_O (x + y likely = 12, Ln = Tb, **1′**; and Eu, **2′**), were prepared by a conventional one-pot reaction (the latter) and recrystallized from DMSO solvent (the former). Polyhydrated intermediates **1′** and **2′** show very similar XRPD patterns, while, despite their common stoichiometry, **1** and **2** are not isostructural. Compound **1** consists of a 2D coordination framework of 3,6 topology, where [Tb(DMSO)_3_]^III^ moieties are bridged by three bis-chelating ClCNAn^2−^ ligands, forming distorted hexagons. Ultrathin nanosheets of **1** were obtained by exfoliation via the liquid-assisted sonication method and characterized by atomic force microscopy, confirming the 2D nature of **1**. The crystal structure of **2**, still showing the presence of 2D sheets with a “hexagonal” mesh and a common (3,6) connectivity, is based onto flat, non-corrugated slabs. Indeed, at a larger scale, the different “rectangular tiles” show clear roofing in **1**, which is totally absent in **2**. The magnetic behavior of **1** very likely indicates depopulation of the highest crystal-field levels, as expected for Tb^III^ compounds.

## 1. Introduction

Lanthanide-based Coordination Polymers (Ln-CPs) and Metal-Organic Frameworks (Ln-MOFs) have attracted considerable interest in material science thanks to their peculiar supramolecular architectures (extending in one, two or three dimensions: 1D, 2D and 3D), their versatile optical properties in the visible (Eu^III^ and Tb^III^) [1,2,3] and near-IR (NIR, for Nd^III^, Er^III^ and Yb^III^) regions, and also their unique magnetic (Ho^III^, Dy^III^ and Tb^III^) properties, due to their intrinsically high magnetic anisotropy, at the origin of interesting magnetic phenomena such as hard magnets or single-molecule magnets. By virtue of these features, they find applications in several applied fields, ranging from telecommunication and data storage to drug delivery, sensing and catalysis [4,5,6,7]. Among these materials, two-dimensional coordination polymers (2D CPs), containing self-assembling metal-ligand-based sheets mutually interacting through weak(er) non-covalent-type interactions as Van der Waals, dipolar and hydrogen-bonding, are of ever-growing interest in material chemistry, as their ultrathin nature favors unique optical, electronic and magnetic properties [8,9,10], as well as chemical processing and rheology, through swelling or exfoliation [11,12].

Among the plethora of ligands which have been employed to fabricate multi-dimensional CPs, 3,6-disubstituted-2,5-dihydroxy-1,4-benzoquinone derivatives, commonly known as anilates, have been widely investigated, by virtue of their Janus-type ability of chelating different metal ions on two opposite sides of the central (and planar) benzoquinone core [13]. The introduction of Lewis-basic functional groups at the 3,6-positions has soon become a viable strategy to avoid the formation of polymeric ribbons, improving material’s dimensionality to sheets of the chess-board type [14]. The resulting 2D materials thus manifested interesting physical properties, as they were found to behave as organic ferroelectrics [15], magnetic conductors [16] or multifunctional MOFs [17]. 

**Chart 1:**
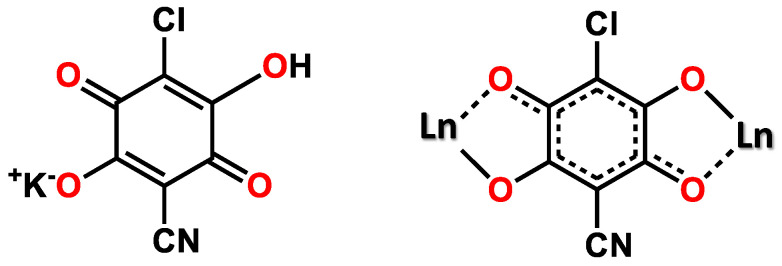
Potassium chlorocyananilate (**left**) and coordination mode of the ligand (**right**).

In 2015, some of us reported on the synthesis and characterization of the first example of a heterosubstituted anilate ligand, 3-chloro-6-cyano-2,5-dihydroxybenzoquinone (in the form of its potassium salt, labeled as KHClCNAn) [18]. In 2018, by using this novel anilate, Gomez-Garcia et al. obtained [Ln_2_(ClCNAn)_3_(solv)_6_]·CPs [Ln^III^ = Ce, Pr, solv = N,N-dimethylformamide (DMF); Yb, Pr, solv = dimethyl sulfoxide (DMSO); Dy, solv = water (H_2_O)] [19] by strictly controlling the reaction conditions (solvent and Ln^III^ size). Furthermore, some of us highlighted the suitability of KHClCNAn to act as an efficient building block for functional (optical and magnetic) 2D CPs and as a sensitizer for NIR lanthanide emission (by the antenna effect). Specifically, by combining a doubly deprotonated ClCNAn^2−^ ligand with Er^III^, Yb^III^ and Nd^III^ ions, 2D [Ln_2_(ClCNAn)_3_(DMF)_6_]·nCH_2_Cl_2_ (Ln = Yb (n = 0), Nd, and Er (n = 2)) CPs, which manifested an efficient energy transfer from triplet states of the ligands to Ln^III^ ions [20], have been prepared. Their 2D structure made this possible by the well-known top-down approach of liquid-assisted exfoliation to ultrathin nanosheets [21,22]. Chart 1 illustrates the basic structural motif present in all these CPs.

In 2019, some of us investigated the structural polymorphism of chlorocyanoanilate-based Dy^III^ CPs. Therein, it was reported how, by changing the synthetic methods (layering technique, solvothermal or conventional one-pot reactions) and conditions (solvent, concentration, etc.), different types of structurally and magnetically characterized 2D extended networks could be selectively obtained. Later, in 2020, Yb^III^-based nanosheets containing two mixed linkers (anilate/carboxylate) were obtained by exfoliation of bulk CPs via the solvent-assisted sonication method [8]. Ultrathin nanosheets were characterized by imaging (atomic force microscopy—AFM and high-resolution transmission electron microscopy—HRTEM) and diffraction (X-ray powder diffraction—XRPD) techniques, highlighting how their optical properties can be affected by the presence of different analytes.

As a further development and investigation of such materials, herein we report the synthesis and structural and magnetic characterization of two 2D CPs, formulated as Ln_2_(ClCNAn)_3_(DMSO)_6_ (Ln^III^ = Tb, **1**; Eu, **2**), which were obtained by recrystallization in DMSO solvent of the related Ln_2_(ClCNAn)_3_(H_2_O)_x_·yH_2_O intermediates (x + y likely = 12, Ln = Tb, **1′**; and Eu, **2′**) synthesized by a one-pot reaction in water.

## 2. Results

**Figure 1:**
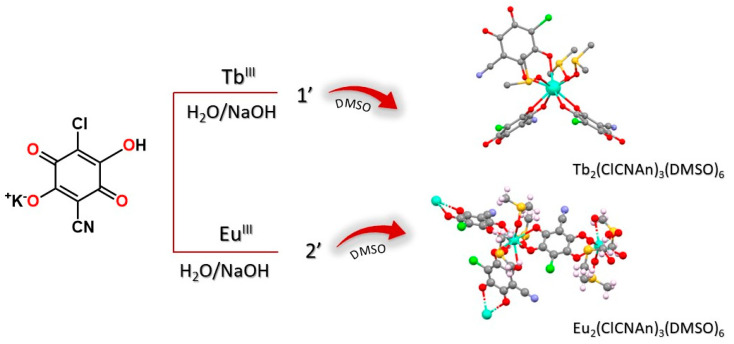
Schematic representation of the synthesis of compounds **1** and **2**, via the intermediacy of **1′** and **2′**.

**Figure 2:**
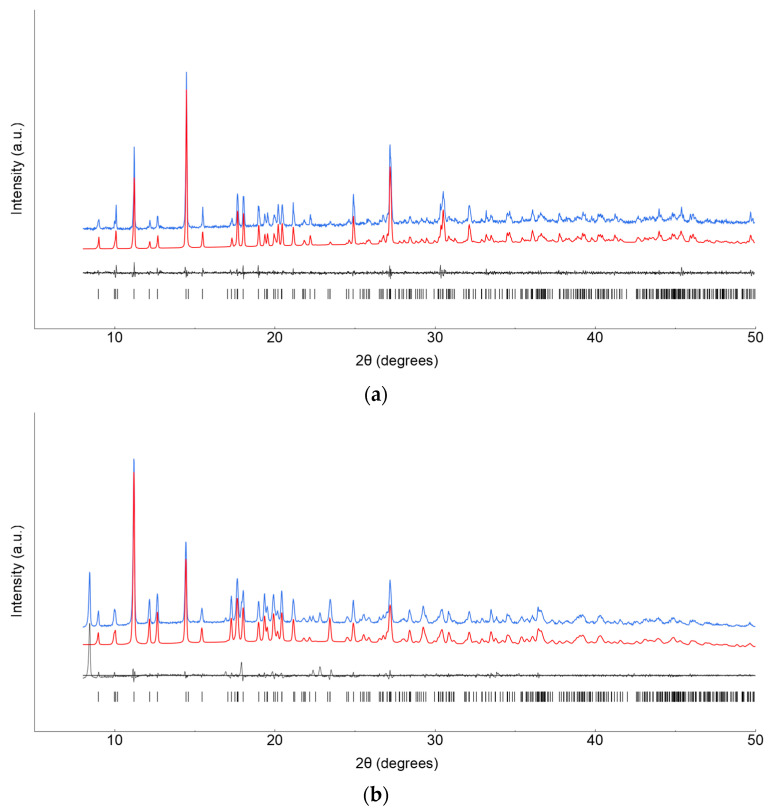
XRPD raw data (blue line) and Le Bail fits (red line) for polycrystalline **1′** (**a**) and **2′** (**b**) species. Difference plot and tick markers are shown in black at the bottom.

Lanthanide ClCNAn^2−^-based polyhydrated compounds **1′** and **2′** are formed by self-assembly of Ln^III^ and chlorocyananilate ions in aqueous solution (Figure 1). Precipitated as reddish polycrystalline powders, they showed very similar XRPD traces (shown in Figure 2a,b, together with their structureless Le Bail fit, obtained after successful indexing of isomorphous unit cells; see Methods and Table 1). Unfortunately, the quality of the diffraction data and the presence of unavoidable contaminant peaks along with the complexity of the material did not enable the determination of the crystal structure and molecular connectivity. This problem was not encountered in the structural determination of the 2D CPs **1** (from conventional single-crystal X-ray diffraction data) and **2** (from PXRD data), later discussed. Notwithstanding, the cell volume of ca. 920 Å^3^ gives a clear indication, following Hofmann’s rules [23], of the material stoichiometry, which we propose to be Ln_2_(ClCNAn)_3_(H_2_O)_x_·yH_2_O, with x + y = 12.

As illustrated in Figure 2, these hydrated species were further recrystallized in DMSO to obtain single crystals of **1**, suitable for X-ray diffraction, and a monophasic polycrystalline material, **2**, characterized by structural PXRD. Eventually, both these species were formulated as Ln_2_(ClCNAn)_3_(DMSO)_6_ (Ln^III^ = Tb for **1** and Eu for **2**).

**Figure 3:**
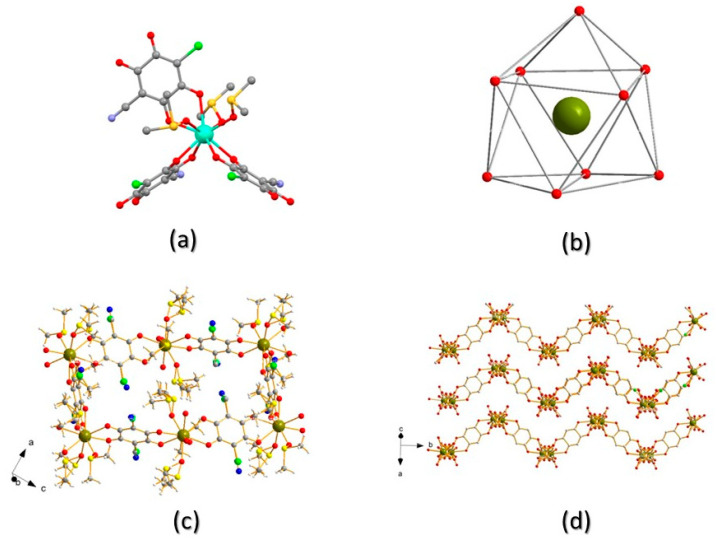
(**a**) Full coordination environment around Tb^III^; (**b**) sketch of the distorted tricapped trigonal prismatic geometry around Tb^III^ (hydrogen and disordered C,S ghosts removed for clarity); (**c**) view of one pseudo-rectangular cavity in ac plane; (**d**) view of three consecutive layers stacking parallel to the (**a**–**c**) vector. Color code: Tb—dark green; O—red; C—gray; S—yellow; Cl—green; N—blue.

Despite their common stoichiometry, compounds **1**, Tb_2_(ClCNAn)_3_(DMSO)_6_, and **2**, Eu_2_(ClCNAn)_3_(DMSO)_6_, are not isostructural and crystallize in the monoclinic P2_1_/n and triclinic P-1 space groups, respectively. Given that the structure of compound **1** has been determined by conventional single-crystal analysis (and not by the less accurate structural powder diffraction methods), the stereochemical description will be mostly focused on **1** (Tb-CP). In this structure, the asymmetric unit contains one Tb^III^ ion, one and a half ClCNAn^2−^ ligands and three DMSO molecules (see Figure 3a). In the ClCNAn^2−^ ligands, chloro and cyano substituents are 50:50 disordered for the (crystallographically imposed) centrosymmetric anilate, and 58:42 for the fully independent ligand. Such a disorder, attributed to residues with similar steric requirements, is indeed commonly observed in ClCNAn^2−^-based CPs [20,24,25].

In addition, the DMSO solvent molecules (apart from the metal-bound oxygen atoms) were found to be severely disordered.

In **1**, the Tb^III^ ion is ennea-coordinated by six oxygen atoms from three different (chelating) ClCNAn^2−^ ligands and by three oxygen atoms from DMSO molecules (Figure 3a), forming a slightly distorted tri-capped trigonal prismatic geometry (Figure 3b). Tb−O bond lengths fall in the 2.332(4)–2.359(4) Å (O=S(CH_3_)_2_) and 2.432(4)–2.483(4) Å (anilate oxygen atoms) ranges, which then cluster into two well-defined classes, differing by ca 0.1 Å. The complex LnO_9_ cage (shown in Figure 3b) shows 36 O-Tb-O bond angles falling in the very wide 63.6(1)–146.6(1)° range, as expected for the very crowded coordination environment of the Tb^III^ ion. Nevertheless, three distinct sets of angles can be envisaged: (i) those near 135° (12×, attributable to six trans cap-Tb-prism and six cap-Tb-cap angles); (ii) those below 90°, for 21 cis O_x_-Tb-O_y_ angles (x, y = cap or prism); and (iii) three intermediate ones (typically > 100°) for the remaining cis O_x_-Tb-O_y_ angles. Two crystallographically distinct (but very similar) Tb···Tb distances are found at 8.807 and 8.825 Å, for terbium atoms bridged by μ_2_,η^4^-chlorocyananilates. These distances are similar to those observed in Dy-ClCNAn_2_-CP (8.167(1) Å) [10]. 

The whole CP structure contains a 2D coordination network (of 3,6 topology, Figure 3c) where three bis-chelating ClCNAn^2−^ ligands bridge [Tb(DMSO)_3_]^3+^ moieties forming distorted hexagons. Indeed, the angles between Tb^III^ ions, taken as the network connecting nodes, are 92.9, 100.5° and 162.9° (2× each, adding up to 712°, witnessing the substantially flat nature of such hexagons—ideal value = (n − 2) × 180°, that is, for n = 6, 720°). These values also offer an estimate of the large deviation of the structure of **2** (the brick-wall type, with 90 and 180° angles) from a regular (3,6) honeycomb structure, where all these angles are exactly 120°, towards degenerate hexagons: that is, rectangles of 2:1 aspect ratios.

In the structure shown in Figure 3d, the corrugated 2D layers (drawn by omitting, for clarity, the disordered Cl/CN and DMSO atoms) are arranged parallel to the (10-1) plane and are highly corrugated, their nominal thickness being ca. 18 Å (the length of the **a**–**c** diagonal). As commonly seen in lanthanide-anilate-based CPs, within these wavy 2D slabs the coordinated DMSO molecules stick out toward the concave potions of the neighboring layers (Figure 3d). A similar structure type was reported for Ln_2_(ClCNAn)_3_(DMSO)_6_ (Ln^3+^ = Dy [10] and Pr [19]).

**Figure 4:**
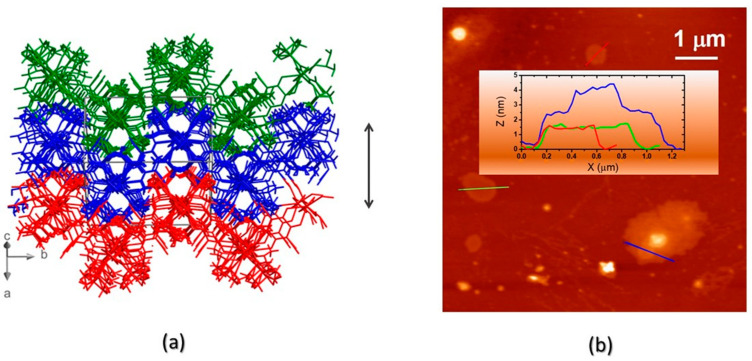
(**a**) The interdigitation of symmetry-related (and heavily corrugated) 2D layers of compound **1**. The orientation of the crystal axes (not to scale) is also shown (bottom left). The double arrow addresses the length of the **a**–**c** vector (18 Å) corresponding to two corrugated layers; (**b**) AFM characterization of drop-casted nanosheets of **1**: topographic image and, in the inset, cross-sectional height profiles, showing that flat or terraced nanosheets are formed, with ca. 9 Å steps.

In Figure 4a, one can appreciate the ca. 18 Å periodicity in the sequence of the layers (2×), which can be used to estimate the number of these slabs (n_s_) within an exfoliated 2D nanocrystal of thickness t (vide infra) as n_s_ = t/9.

Morphological characterization of the corresponding nanosheets of **1** CP was performed by AFM, on drop-casted suspensions, obtained by crystal sonication, confirming the 2D nature of **1** bulk size CP. Remarkably, micrometer-sized nanosheets were obtained, with heights ranging from one to four layers, as clearly shown in Figure 4b. 

**Figure 5:**
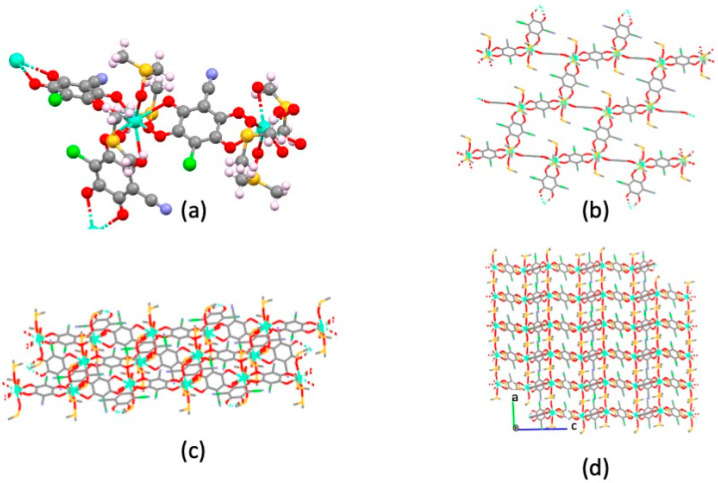
Sketches of the main structural features of **1** CP. (**a**) A fragment of the CP, showing the local environment of the Eu^III^ ions; (**b**,**c**) the brick-wall connectivity of a flat slab in **2**, viewed down the axis normal to the CP extension, and from the side, respectively; (**d**) the overall crystal packing viewed down the **a** axis, showing that slabs, interacting only through weak van der Waals contacts mostly attributed to the DMSO ligands, stack along **b** with a ca. 9.7 Å periodicity.

The structure of compound **2**, determined (in the absence of single crystal specimens of suitable size and quality) by unconventional powder diffraction methods, is here discussed only from a connectivity and topological point of view. Indeed, as several restraints were added to stabilize convergence to physically meaningful results, with an extensive usage of rigid bodies to describe the chlorocyananilate and DMSO ligands, no substantial stereochemical (bond distances and angles) descriptors can be taken as being accurate enough. Nevertheless, the following discussion does not suffer from such inaccuracy, and includes fully reliable (packing) features. In the crystal structure of **2**, the asymmetric unit contains one Eu^III^ ion, three distinct half ClCNAn^2−^ ligands and three DMSO molecules (see Figure 5a for a slightly larger fragment, excised from the whole structure). All being chlorocyananilate ligands located onto three different inversion centers, they all have crystallographically imposed disordered 50:50 Cl/CN residues. Also, in compound **2**, the lanthanide ion is ennea-coordinated, with three chelating chlorocyananilates and three individual O-bound DMSO molecules.

Differently from the structure of **1**, the overall crystal structure of Eu_2_(ClCNAn)_3_(DMSO)_6_, still showing the presence of 2D sheets with a “hexagonal” mesh and a common (3,6) connectivity, is based onto flat, non-corrugated slabs (as per Figure 5b,c). The internal angles of these degenerate hexagons are 88.1, 109.5 and 156.9° (2×, adding up to 709°). All very similar to those found in compound **1** and presented above, the interionic Eu⋯Eu separations of 8.83, 8.84 and 8.89 Å alone do not provide any direct hint of the significantly different warping of the 2D slabs in **1** and **2**. Indeed, the differences arise at a larger scale, where the different “rectangular tiles” show clear roofing in **1**, which is totally absent in **2** (dihedral angles between tiles of 102.1° and 0°, respectively).

**Figure 6:**
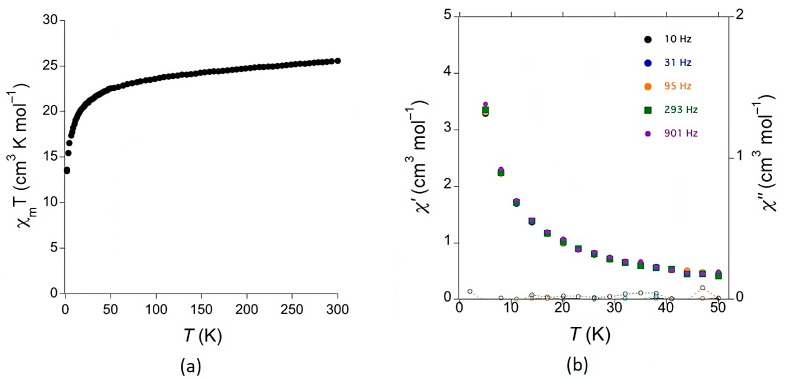
(**a**) Plot of the χmT product as a function of temperature for **1**. (**b**) In-phase (χ′) and out-of-phase (χ″) dynamic susceptibility for **1** at different frequencies as a function of temperature.

Magnetic measurements were carried out with a fine powder sample of **1** as a function of temperature (Figure 6). The χ_m_T value at room temperature (25.1 cm^3^ K mol^–1^) is in good agreement with the expected value for two magnetically independent Tb^III^ cations. The high spin–orbit coupling found in rare earths is responsible for such a high magnetic moment, arising from a ground state defined by J = 6. For a theoretical g = 3/2, a χ_m_T ≈ 11.81 cm^3^ K mol^–1^ is expected [26], but room temperature χ_m_T products up to ≈ 13 cm^3^ K mol^–1^ have been reported for a single Tb^III^ cation [27]. Also expected for Tb^III^, the χ_m_T value decreases when the temperature is decreased, because of the depopulation of the higher crystal-field levels. The high anisotropy of the Tb^III^ centers does not allow detection of any additional super-exchange interactions promoted by the bridging organic ligands. Dynamic (AC) magnetic susceptibility measurements were performed down to 2 K at different frequencies (Figure 6). No out-of-phase χ″ signal was observed, not even when an additional DC magnetic field was applied, indicating no SMM (Single Molecular Magnet) behavior for this compound.

## 3. Conclusions

The ditopic capability of chlorocyanoanilate ligands to chelate metal ions on two opposite sides of a central (and planar) benzoquinone core has been successfully exploited in the construction of 2D lanthanide coordination polymers formulated as Ln_2_(ClCNAn)_3_(DMSO)_6_ (Ln^III^ =Tb for **1** and Eu for **2**), generated from corresponding hydrated intermediates. Remarkably, the polyhydrated intermediates are isostructural, while recrystallization from DMSO by slow evaporation affords **1** and **2**, which are not isostructural despite their common stoichiometry and crystallize in the monoclinic P2_1_/n and triclinic P-1 space groups, respectively. A single-crystal X-ray study of **1** shows that the Tb^III^ ion is ennea-coordinated within a slightly distorted tri-capped trigonal prismatic geometry, where bis-chelating ClCNAn^2−^ ligands bridge [Tb(DMSO)_3_]^3+^ moieties, providing 2D corrugated layers. The 2D character of the coordination network in **1** allowed its exfoliation into nanosheets imaged by AFM. The magnetic susceptibility measurements of **1** are in agreement with isolated Tb^III^ centers without slow magnetic relaxation. Despite their high magnetic anisotropy, the appearance of SMM behavior in rare earth complexes is fully dependent on the geometry imposed by the ligands, which is difficult to predict or tune in the solid state structure, especially with flexible organic ligands/linkers [28]. The structure of the Eu^III^ coordination polymer **2**, as determined from PXRD measurements, is slightly different compared to that of **1** since the slabs are not corrugated. These results nicely complete and complement the series of coordination polymers based on chlorocyananilate ligands and lanthanide ions. Variation of the anilate substituents and lanthanides co-ligands is envisaged in order to tailor the magnetic and optical properties of these 2D materials.

## 4. Materials and Methods

Materials Reagents were purchased from Zentek (TCI) and used without further purification. HPLC-grade solvents were purchased from Thermofisher Scientific Alfa-Aesar. KHClCNAn was synthesized as reported in literature [18]. 

*Synthesis of Tb_2_(ClCNAn)_3_(DMSO)_6_*. (**1**). An aqueous solution of Tb(NO_3_)_3_·5H_2_O (0.30 mmol; 103 mg) was added dropwise to an aqueous red solution of KHClCN (0.15 mmol; 36 mg) and NaOH (0.10 mmol; 7.2 mg), showing an immediate color change to purple. After stirring at 90 °C for ca. 4 h, a purple precipitate appeared (**1′**). The mixture was cooled down to room temperature and the powder was collected from the mother liquor by vacuum filtration. The solid was then washed several times with cold deionized water. 

Analytical evidence suggested a Tb_2_(ClCNAn)_3_(H_2_O)_x_ chemical formula for this intermediate (**1′**). Red prismatic crystals of **1**, suitable for X-ray analysis, were obtained by recrystallization from DMSO (15 mg of **1′** in 10 mL of DMSO) by slow evaporation, in a petri vial, at room temperature (T = 25 °C), within one week.

*Synthesis of Eu_2_(ClCNAn)_3_(DMSO)_6_.* (**2**) This compound was synthesized with a similar synthetic approach, using Eu(NO_3_)_3_·5H_2_O instead of Tb(NO_3_)_3_·5H_2_O. As for the above synthesis, the **2′** and **2** labels are associated with a red hydrated polycrystalline intermediate and DMSO-containing recrystallized polycrystalline material, respectively.

*Synthesis of Nanosheets of **1**.* Nanosheets were fabricated using the top-down sonication-assisted exfoliation method. Delamination was achieved by sonicating (Bandelin electronic equipment at 230 V) the dried powder of **1** CPs (1 mg) samples in isopropanol (1 mL) for 15 min at room temperature.

X-ray Single-Crystal Structure Determination. A single crystal of compound **1** was mounted on a glass fiber loop using a viscous crystal-coating hydrocarbon oil and was immediately transferred to the diffractometer cradle equipped with a cold N_2_ stream. Data collection was performed at 150 K on an Agilent Supernova Diffractometer with monochromatized Cu Kα radiation (λ = 1.54184 Å). The structure was solved by direct methods with the SIR97 program [29] and refined against all F^2^ values using the SHELXL-97/ WinGX suite of programs [30]. All H atoms were placed in calculated positions and refined isotropically with a riding model. Non-H atoms were refined anisotropically except for the disordered ones: C7, C8 and C12 within the cyano groups and the methyl residues of the DMSO molecules. Crystallographic data and refinement parameters for **1** are listed in Table 2. Full crystal data, in the standard Crystallographic Information File format, have been deposited at the Cambridge Crystallographic Data Centre (CCDC Code: 2284151).

X-ray Powder Diffraction-Crystal Structure Determination. Samples of **1′**, **2′** and **2** were gently ground in an agate mortar and then deposited in the hollow of a silicon monocrystal zero-background plate (supplied by Assing SpA, Monterotondo, Italy). XRPD measurements were performed using a Bruker AXS D8 Advance diffractometer in Bragg-Brentano θ:θ geometry, equipped with a Lynxeye position sensitive detector. DS: 0.5°; generator setting: 40 kV, 40 mA; Ni-filtered Cu-Kα radiation, λ = 1.5418 Å, 2θ-range: 3–50°. XRPD data for structure solution and refinement of species **2** were collected in the 3–105° 2θ range, sampling at 0.02°, with scan time lasting approximately 16 h.

### 4.1. Cell Determination from X-ray Diffraction Data

Standard peak search methods followed by the accurate estimate of the low-angle peak position and the use of the singular value decomposition protocol [31] implemented in TOPAS-R (V.3.0, 2005, Bruker AXS, Karlsruhe, Germany) enabled the detection of triclinic unit cells with GOF(20) = 26.1 and 61.5, for **1′** and **2**, respectively. The structureless Le Bail whole pattern profile fitting method was used to refine the lattice parameters of the isomorphous **1** and **2** species, evidencing the presence of unavoidable contaminants (perhaps differently hydrated species). Therefore, no structure solution attempt was found to be successful in retrieving a suitable model.

### 4.2. Ab Initio Crystal Structure Solution from X-ray Diffraction Data

An XRPD structure solution of the species **2** phases was performed in space group P-1 using TOPAS-R software with the Monte Carlo/Simulated Annealing technique using a single Eu^3+^ ion, rigid models for ClCNAn^2−^ and DMSO ligands described by the Z-matrix formalism with standard geometrical parameters. It was eventually found that all chlorocyananilate ligands lie on inversion centers with consequent 50:50 Cl/CN disorder. Due to the less-than-ideal quality of the XRPD data, no attempt to determine (static or dynamic) disorder of the DMSO molecules was made.

**Figure 7:**
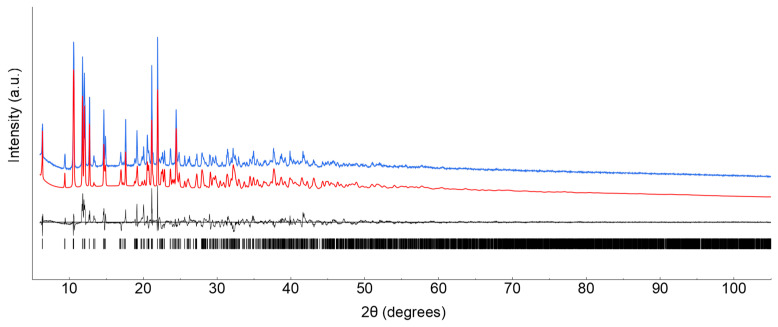
Final Rietveld refinement plot for species **2**. Observed data in blue; calculated data in red. The difference plot (in black) and tick markers for peak positions are shown at the bottom.

Final refinements were eventually carried out by the Rietveld method [32], maintaining the rigid bodies introduced at the structure solution stage and the crystallographically imposed symmetries. The background was modelled by a polynomial function of the Chebyshev type; peak profiles were described by the Fundamental Parameters Approach [33] and a common (refinable) isotropic thermal factor was attributed to all atoms. Fractional atomic coordinates and crystal structure details are supplied in the Supplementary Materials. The final Rietveld plot is shown in Figure 7.

Magnetic Measurements Magnetic measurements were carried out using a Quantum Design MPMS-XL magnetometer. Magnetic susceptibility data were recorded in the 2–300 K temperature range in an external field of 1000 Oe. Diamagnetic corrections were calculated using Pascal’s constants. Dynamic magnetic susceptibility data were obtained in the frequency range of 0.1–1000 Hz in an oscillating field of 3 Oe.

AFM characterization NT-MDT Solver-Pro atomic force microscopy (AFM) was used to study the topography and roughness of the nanosheets. AFM measurements were performed at 0.5–1 Hz scan speed in semicontact mode in air. Topographic image analysis and calculation of surface roughness were performed using WSxM 5.0 Develop3.2 software.

## Figures and Tables

**Table 1:** Crystal data for compounds Ln_2_(ClCNAn)_3_(H_2_O)_x_·y(H_2_O) x + y = 12 (Ln = Tb, **1′**; Eu, **2′**).

Species	Symmetry	a, Å	b, Å	c, Å	α, °	β, °	γ, °	V, Å^3^
**1′**	Triclinic	10.12	10.68	10.36	73.3	88.0	60.0	920.7
**2′**	Triclinic	10.13	10.70	10.36	73.4	88.0	59.9	922.5

**Table 2:** Crystallographic data and details of the refinement procedure for compounds **1** and **2**.

Compound	(1)	(2)
Formula	C_33_H_36_Cl_3_N_3_O_18_S_6_Tb_2_	C_33_H_36_Cl_3_Eu_2_N_3_O_18_S_6_
Few	1379.20	1365.28
Crystal color	Red	Red
Sample size (mm^3^)	0.02 × 0.04 × 0.07	0.02 × 8.0 × 10.0
Temperature (K)	150 (2)	298 (2)
Wavelength (Å)	1.54184	1.54184
Crystal system, Z	Monoclinic, 2	Triclinic, 1
Space group	P2_1_/n	P-1
a (Å)	9.6868 (2)	9.704 (1)
b (Å)	16.3511 (3)	9.710 (1)
c (Å)	15.1558 (3)	14.146 (1)
α (°)	90	84.003 (6)
β (°)	93.683 (2)	97.145 (6)
γ (°)	90	78.526 (5)
V (Å^3^)	2395.57 (8)	1284.6 (4)
ρ_calc_ (g.cm^−3^)	1.912	1.764
μ(CuKα) (mm^−1^)	18.94	19.92
θ range (°)	3.981–73.587	2.5–52.5
Data collected	19515	5001
Data unique	4767	-
Data observed	4408	-
Number of parameters/restraints	388/65	58/9
R(int)	0.0486	-
R1(F), ^a^ I > 2σ(I)	0.0490	(Rp) 0.0491
wR2(F^2^), ^b^ all data	0.1253	(Rwp) 0.0755
S(F^2^), ^c^ all data	1.106	6.13

^a^ R1(F) = ΣǁFo| − |Fcǁ/Σ|Fo|; ^b^ wR2(F^2^) = [Σw(Fo^2^ − Fc^2^)^2^/ΣwFo^4^]^1/2^; ^c^ S(F^2^) = [Σw(Fo^2^ − Fc^2^)^2^/(n + r − p)]^1/2^.

## Data Availability

Not applicable.

## Supplementary Materials

The following are available online at https://www.mdpi.com/article/10.3390/molecules28186453/s1, Figure S1: AFM topographic image showing exfoliation of **1;** Table S1: Crystal Data for compound **2**.
