# Supplementary material for: Terbium and Europium Chlorocyananilate-Based 2D Coordination Polymers
Source: Molecules. 2023 Sep 6;28(18):6453. doi: 10.3390/molecules28186453 (PMC10535540; doi:10.3390/molecules28186453)
Supplement: Supplementary file 1 [file molecules-28-06453-s001.zip › molecules-2565778-supplementary.pdf]

Terbium and Europium Chlorocyananilate-Based 2D Coordination Polymers

Mariangela Oggianu [1,2], Alexandre Abhervé [3], Daniela Marongiu [4], Francesco Quochi [2,4], José Ramón Galán-Mascarós [5,6], Federica Bertolotti [7], Norberto Masciocchi [2,7], Narcis Avarvari [3,*] and Maria Laura Mercuri [1,2,*]

1 Department of Chemical and Geological Sciences, University of Cagliari, Highway 554, Crossroads for Sestu, I-09042 Monserrato, Italy; mariangela.oggianu@unica.it
2 National Interuniversity Consortium of Materials Science and Technology, INSTM, Street Giuseppe Giusti, 9, I-50121 Florence, Italy
3 Laboratoire MOLTECH-Anjou UMR 6200, UFR Sciences, CNRS, Université d'Angers, Bat. K, 2 Bd. Lavoisier, I-49045 Angers, France; alexandre.abherve@univ-angers.fr
4 Department of Physics, University of Cagliari, Highway 554, Crossroads for Sestu, I-09042 Monserrato, Italy; dmarongiu@dsf.unica.it; quochi@unica.it (F.Q.)
5 Institute of Chemical Research of Catalonia (ICIQ-CERCA), Barcelona Institute of Science and Technology (BIST), Avenida Països Catalans 16, 43007 Tarragona, Spain; jrgalan@iciq.es
6 ICREA, Av. Lluis Companys 16, *Passeig* Lluís Companys, 23, 08010 Barcelona, Spain
7 Dipartimento di Scienza e Alta Tecnologia & To.Sca.Lab., University of Insubria, Via Valleggio 11, I-22100 Como, Italy; federica.bertolotti@uninsubria.it (F.B.); norberto.masciocchi@uninsubria.it (N.M.).
* Correspondence: narcis.avarvari@univ-angers.fr (N.A.); mercuri@unica.it (M.L.M.); Tel.: +33-241-735-084 (N.A.); +39-070-675-4474 (M.L.M.); Fax: +33-02-41-73-5405 (N.A.); +39-070-675-4486 (M.L.M.)

Table S1.

Crystal data for compound 2, $Eu_2(ClCNAn)_3(DMSO)_6$, derived from XRPD analysis.

Cell a = 9.704(1), b = 9.710(1), c = 14.146(1) Å,
α = 84.003(6), β = 97.145(6), γ = 78.526(5) °

Cell Volume: V = 1284.6(4) Å^3

Crystal System: Triclinic

Space Group: P-1

Fractional atomic coordinated for compound 4, $Eu_2(ClCNAn)_3(DMSO)_6$, derived from XRPD analysis.

site	Eu	x	0.67059	y	0.76545	z	0.27392
site	C1	x	-0.07873	y	0.38790	z	0.50553
site	C2	x	-0.12233	y	0.50257	z	0.43207
site	C3	x	-0.03870	y	0.62163	z	0.42620
site	O1	x	-0.22912	y	0.50832	z	0.37124

site O2 x -0.07763 y 0.72401 z 0.36061
site C7 x -0.15747 y 0.27580 z 0.51105
site N1 x -0.21991 y 0.18690 z 0.51544
site Cl1 x -0.17376 y 0.25261 z 0.51220

site C1a x 0.61443 y -0.09135 z 0.56767
site C2a x 0.52954 y 0.02603 z 0.59701
site C3a x 0.40801 y 0.12304 z 0.52513
site O1a x 0.55240 y 0.05208 z 0.68153
site O2a x 0.33221 y 0.22784 z 0.55132
site C7a x 0.72887 y -0.18269 z 0.63534
site N1a x 0.81963 y -0.25514 z 0.68902
site Cl1a x 0.75254 y -0.20159 z 0.64935

site C1b x 0.63944 y 0.01629 z -0.01963
site C2b x 0.51787 y 0.08608 z -0.08370
site C3b x 0.36977 y 0.06878 z -0.06285
site O1b x 0.52953 y 0.16243 z -0.15783
site O2b x 0.26122 y 0.13109 z -0.12006
site C7b x 0.77889 y 0.03258 z -0.03926
site N1b x 0.88948 y 0.04550 z -0.05483
site Cl1b x 0.80774 y 0.03595 z -0.04332

site O01 x 0.50419 y 0.37178 z 2.70007
site S1 x 0.60409 y 0.49576 z 2.71327
site C01 x 0.52443 y 0.61892 z 2.78177
site C02 x 0.77372 y 0.41248 z 2.77096
site H011 x 0.51888 y 0.57145 z 2.84295
site H012 x 0.58026 y 0.68821 z 2.78915
site H013 x 0.43168 y 0.66280 z 2.74975
site H021 x 0.81548 y 0.34496 z 2.73310
site H022 x 0.82954 y 0.48176 z 2.77833
site H023 x 0.76658 y 0.36632 z 2.83221

site O11 x -3.70193 y 2.40385 z 0.83920
site S2 x -3.85967 y 2.43946 z 0.88188
site C11 x -3.97327 y 2.58137 z 0.80738
site C12 x -3.93354 y 2.29014 z 0.88297
site H111 x -3.98777 y 2.55674 z 0.74420
site H112 x -4.06142 y 2.60127 z 0.83123
site H113 x -3.93119 y 2.66176 z 0.80698
site H121 x -3.87002 y 2.21339 z 0.92336
site H122 x -4.02169 y 2.31005 z 0.90682
site H123 x -3.94829 y 2.26737 z 0.81930

site O21 x -5.27933 y 1.98208 z 0.28941

site	S3	x	-5.12803	y	2.04603	z	0.29802
site	C21	x	-5.04815	y	2.01645	z	0.41759
site	C22	x	-5.01344	y	1.95861	z	0.22756
site	H211	x	-5.02605	y	1.91743	z	0.43887
site	H212	x	-4.96360	y	2.05219	z	0.42240
site	H213	x	-5.11221	y	2.06396	z	0.45524
site	H221	x	-5.05876	y	1.97490	z	0.16268
site	H222	x	-4.92889	y	1.99435	z	0.23238
site	H223	x	-4.99156	y	1.85996	z	0.25006

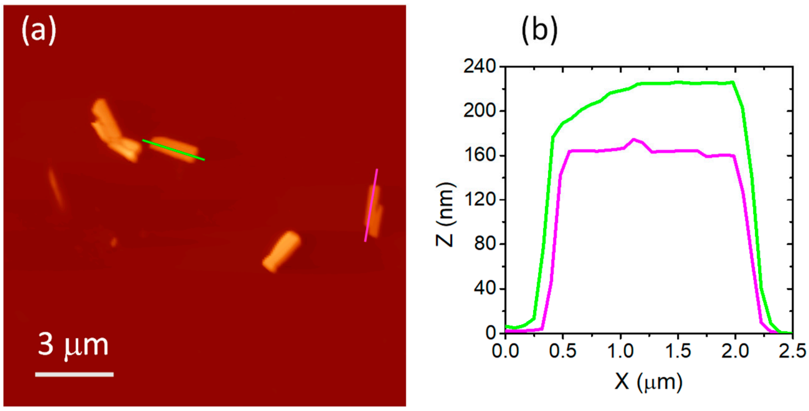

Figure S1. (a) AFM topographic image showing exfoliation products of **1** with heights ranging in the hundreds of nanometers. (b) Height profiles obtained along the lines drawn in panel (a).
